# Endosomal Cholesterol in Viral Infections – A Common Denominator?

**DOI:** 10.3389/fphys.2021.750544

**Published:** 2021-11-11

**Authors:** Mirco Glitscher, Eberhard Hildt

**Affiliations:** Department of Virology, Paul-Ehrlich-Institute, Langen, Germany

**Keywords:** cholesterol, trafficking, viruses, endosomes, antivirals, host-factors

## Abstract

Cholesterol has gained tremendous attention as an essential lipid in the life cycle of virtually all viruses. These seem to have developed manifold strategies to modulate the cholesterol metabolism to the side of lipid uptake and *de novo* synthesis. In turn, affecting the cholesterol homeostasis has emerged as novel broad-spectrum antiviral strategy. On the other hand, the innate immune system is similarly regulated by the lipid and stimulated by its derivatives. This certainly requires attention in the design of antiviral strategies aiming to decrease cellular cholesterol, as evidence accumulates that withdrawal of cholesterol hampers innate immunity. Secondly, there are exceptions to the rule of the abovementioned virus-induced metabolic shift toward cholesterol anabolism. It therefore is of interest to dissect underlying regulatory mechanisms, which we aimed for in this minireview. We further collected evidence for intracellular cholesterol concentrations being less important in viral life cycles as compared to the spatial distribution of the lipid. Various routes of cholesterol trafficking were found to be hijacked in viral infections with respect to organelle-endosome contact sites mediating cholesterol shuttling. Thus, re-distribution of cellular cholesterol in the context of viral infections requires more attention in ongoing research. As a final aim, a pan-antiviral treatment could be found just within the transport and re-adjustment of local cholesterol concentrations. Thus, we aimed to emphasize the importance of the regulatory roles the endosomal system fulfils herein and hope to stimulate research in this field.

## Introduction

Viral infections certainly present one of the biggest concerns of our society leading to a tremendous number of deaths worldwide. In the fight against virus-induced diseases, several antiviral medications have emerged and were approved (de Clercq and Li, 2016). However, the majority of these compounds are either specific for a class of viruses, induce heavy adverse effects, or lead to viral escape mutations (Sleijfer et al., 2005; de Clercq and Li, 2016; Domingo et al., 2019). Thus, a search for a tolerable and pan-antiviral therapy has emerged. A strong focus now is set on affecting the host’s cholesterol metabolic network (Lembo et al., 2016; Yuan et al., 2019; Gorabi et al., 2020). For reasons explained in this article, a variety of studies focus on the reduction of cellular cholesterol. Recent evidence implies that this may not only present a strategy against enveloped viruses. Various non-enveloped viruses make use of the exosomal pathway or autophagy for their release, thus being dependent on membranous processes just as well (Jiang et al., 2011; Zhang et al., 2021). Consequently, the majority of studies follow the assumption that a certain net amount of cellular cholesterol *per se* is required in viral life cycles. While this may be true for various viruses, some examples demonstrated that cholesterol depletion actually behaves provirally (Jung et al., 2012; Blanchet et al., 2016; Glitscher et al., 2021). While altering the cholesterol homeostasis in the host appears as a promising pan-antiviral strategy, ambiguous behaviors hinder the progress in finding exactly this. A problem therefore addressed by us is that it may not come down to how much cholesterol resides within a cell. Rather, we believe, it is important where and how the cholesterol pool is distributed within a cell. We therefore try to give an overview of the current knowledge, gaps in knowledge, and possible prospects in how cholesterol trafficking mediated by endosomes may be useful to this overall aim.

## Modulation of Cellular Cholesterol Content in Viral Infections

### Cholesterol Synthesis and Uptake

A wide range of viruses induces cholesterol anabolism, suggesting a general shift toward cholesterol uptake and *de novo* synthesis in infected cells. Central processes hereof are transcriptionally regulated by sterol-regulatory element binding proteins (SREBPs). These ER-resident proteins are maintained in a complex together with insulin-induced gene 1 (INSIG1), SREBP cleaving-activating protein (SCAP), and sterols (Yang et al., 2002). Upon depletion of the latter, INSIG1 dissociates and the SREBP-SCAP complex traffics to the Golgi apparatus (Sun et al., 2007). Subsequent cleavage releases the transcription factor (TF) domain to be shuttled into the nucleus (Wang et al., 1994). Herein, SREBPs drive the expression of a variety of genes being responsible for increasing cellular cholesterol. Two major key players are the 3-hydroxy-3-methyl-glutaryl-coenzyme A reductase (HMGCR), catalyzing the rate-limiting step in cholesterol synthesis (Geelen et al., 1986), and the low-density lipoprotein (LDL) receptor (Yokoyama et al., 1993), binding the major cholesterol-carrying lipoprotein. This point of control is hijacked by a variety of viruses. Human cytomegalovirus (HCMV), hepatitis B virus (HBV) or hepatitis C virus (HCV) activate SREBP activity through enhancing its proteolytic cleavage (Waris et al., 2007; Yu et al., 2012; Qiao et al., 2013). Additionally, a correlation between early viral infections and a rise in LDL uptake and cholesterol biosynthesis were observed for Dengue virus (DENV) or West Nile virus (WNV; Mackenzie et al., 2007; Soto-Acosta et al., 2013). Also for non-enveloped viruses, such as the Coxsackievirus B3 (CVB3), an activation of SREBPs was observed (Wang et al., 2018) and may be similarly important for viruses making use of the exosomal pathway, such as Enterovirus 71 (EV71), human Norovirus (HuNoV), Rotaviruses, or encephalomyocarditis virus (EMCV; Owusu et al., 2021). Following these findings, cholesterol removal from infected cells could correlate with benefits for a viral infection.

### Cholesterol Export

On the other hand, cholesterol depleting processes need to be taken into account, which are majorly regulated *via* two different nuclear receptors acting as TFs: (i) liver X receptors (LXR) and (ii) farnesoid X receptors (FXR). The former is activated by sterols and oxysterols (Janowski et al., 1996) and primes expression of the cholesterol export program. Among various targets, ATP-binding cassette transporter A1 (ABCA1) is induced, which shuttles cholesterol into the extracellular space. This process is aided by cholesterol transport from endolysosomes to the plasma membrane (PM) *via*, for example, the Niemann-Pick C1 protein (NPC1; Boadu and Francis, 2006; Boadu et al., 2012). As a result, high-density lipoprotein (HDL) is formed with the help of ATP-binding cassette transporter G1 (ABCG1) and apolipoprotein A1 (ApoA1) leading to cholesterol export from cells (Gelissen et al., 2006; Zhao et al., 2012). At this level, viruses, such as human immunodeficiency virus (HIV), HCV, and HCMV, have been demonstrated to impair ABCA1 activity *via* reduced expression (Mujawar et al., 2006; Sanchez and Dong, 2010; Shirasaki et al., 2013). Further, both HBV and severe acute respiratory syndrome coronavirus 2 (SARS-CoV-2) seem to decrease ApoA1 levels (Wang et al., 2016; Yang et al., 2020), thereby also reducing HDL-related cholesterol export. Effectively, these regulations would match to an increase in cholesterol anabolism, thus increasing the lipid in an infected cell.

### Cholesterol Derivatization

Apart from cholesterol export, derivatization into bile acids (BAs) is a way to rid a cell from the lipid specifically in liver tissue. The expression of both rate-limiting enzymes, namely, the ER-resident cholesterol 7 alpha-hydroxylase (CYP7A1) or the mitochondrial sterol 27-hydroxylase (CYP27A1), is induced by LXR (Gupta et al., 2002; Szanto et al., 2004). Once primary bile acids are synthesized, FXR represses these anabolic enzymes (Forman et al., 1995; Chiang, 2015) and enhances BA export *via* ATP-binding cassette transporter B11 (ABCB11; Ananthanarayanan et al., 2001; Deng et al., 2006) or the organic solute transporter subunit alpha and beta (OSTα/β; Ballatori et al., 2005; Landrier et al., 2006). Both LXR and FXR activity is fine-tuned by co-factors represented by the nuclear receptors retinoic X receptors (RXR) or peroxisome proliferator-activated receptors (PPARs; Seol et al., 1995; Kassam et al., 2003; Pineda Torra et al., 2003). As a liver-specific process, BA synthesis and export are mainly affected by enteric and hepatotropic viruses. For instance, an activation of FXR is related to proviral effects for HBV, HCV, or HuNoV (Scholtes et al., 2008; Mouzannar et al., 2019; Murakami et al., 2020). In turn, the activation of FXR would result in an impaired BA synthesis and render cholesterol levels to rise. This again supports the claim of viruses elevating cellular cholesterol.

### Cholesterol Storage

A third way of removing cholesterol from a cell is *via* esterification and subsequent intracellular storage. This catalytic process is mediated by the ER-resident acyl-coenzyme A:cholesterol acyltransferase (ACAT), which is activated by cholesterol (Cheng et al., 1995). As a consequence, cholesterol esters are stored in lipid droplets (LDs) rendering cholesterol to be biologically inactive. The morphogenesis and depletion of LDs are strictly coordinated (Olzmann and Carvalho, 2019) and similarly are made use of by different viruses. The predominant example is HCV, which strongly depends on these structures for virion morphogenesis (Barba et al., 1997; Boulant et al., 2007). However, also a variety of other viruses induce the formation of LDs among which are DENV, Zika virus (ZIKV), Herpes simplex virus 1 (HSV-1), Influenza A virus (IAV; Samsa et al., 2009; Monson et al., 2021), and Adenovirus RIDα (Cianciola et al., 2013). Consequently, viruses requiring LDs present a group contradicting with a general increase in biologically available net cholesterol content in cells. While they do require cholesterol synthesis and uptake, it would then be stored rather than being available within membranes.

## Innate Immunity

### Pathogen Recognition Receptors

Just as viral life cycles, the innate immune response against the pathogens is regulated by cholesterol, which is initiated by pathogen-associated molecular patterns (PAMPs) being sensed by pathogen recognition receptors (PRRs). Here, toll-like receptors (TLRs) represent PRRs being either present on the PM or within endosomes majorly recognizing nucleic acids (Latz et al., 2004; Nishiya et al., 2005; Husebye et al., 2006; Johnsen et al., 2006; Nilsen et al., 2008; Ishii et al., 2014; Lester and Li, 2014). They share a close relationship with ABCA1-dependent cholesterol removal, which interferes with downstream cascades and vice versa (Zhu et al., 2008, 2010; Guo et al., 2015; Li et al., 2018; Ding et al., 2020). Similarly, TLR activity seems to decline upon application of statins, inhibitors of cholesterol synthesis (Mullen et al., 2015; Bahrami et al., 2018; Venardos et al., 2018). A second class of PRRs is represented by retinoic acid-inducible gene-I-like receptors (RIG-I-like receptors or RLRs). An activation by PAMPs subsequently triggers oligomerization of mitochondrial antiviral signaling protein (MAVS) on the outer mitochondrial membrane, which passes signaling on to the production of IFNs (Zamorano Cuervo et al., 2018; Rehwinkel and Gack, 2020). While little is known about cholesterol controlling the related pathway, RIG-I signaling seems to involve the oxysterol 25-hydroxycholesterol (Wang et al., 2012). Further, the predominant localization of MAVS at ER-mitochondrion contact sites with high cholesterol content (Horner et al., 2011) implies that its oligomerization could be regulated by local cholesterol concentrations. Further elucidation is required to understand these relationships in detail.

### Interferon Response

Downstream of PRRs, respective signaling cascades elicit the production of cytokines among which are IFNs. These result in myriads of regulatory alterations in targeted cells, ultimately mediating host-defense mechanisms. An interesting link to cholesterol can be drawn by IFNs inducing a key enzyme in the buildup of cholesterol-derivatives, namely, cholesterol 25-hydroxylase (CH25H). The latter produces an oxysterol, 25-hydroxycholesterol, which has been described as broadly antiviral *via* diverse mechanisms (Zhao et al., 2020) against, for example, HSV, IAV, SARS-CoV-2, and members of the *Flaviviridae* or *Filoviridae* families (Liu et al., 2013; Chen et al., 2014; Wang et al., 2020). In general, oxysterols also serve important functions in regulating the IFN response and production of the cytokines. Oxysterol production is inducible by IFNs (Marquis et al., 2011; Blanc et al., 2013).These in turn activate LXR (Lehmann et al., 1997), which boosts production of IFNγ (Wang Q. et al., 2014). This could serve the purpose of inducing antiviral activity, as LXR has been described to induce viral restriction if induced pharmacologically (Sheng et al., 2016; Lange et al., 2018, 2019; Mlera et al., 2021). Further, oxysterols themselves exert similar antiviral effects against manifold viruses (Civra et al., 2014, 2018; Anggakusuma et al., 2015; Willard et al., 2018; Marcello et al., 2020; Zu et al., 2020; Glitscher et al., 2021). Opposing to this, however, stand studies describing a negative feedback loop of IFNγ reducing LXR and FXR activity (Renga et al., 2009; Pascual-García et al., 2013). Effectively, this would increase cellular cholesterol levels, as observed in further studies (Hao et al., 2013; Robertson and Ghazal, 2016; Kühnl et al., 2018). More research is required elucidating the mechanism regulating these opposing effects especially with respect to the change in net cholesterol, which previously was assumed to be essential to viruses.

### Inflammasome

The inflammasome is a multiprotein complex and a prime mediator for inflammatory processes and pyroptosis. Upon establishment of cellular stress or exposure to pathogens and subsequent PAMP-sensing, different subunit combinations of the complex assemble. In its core, homo-oligomers of either pro-caspase 1 or 8 are found. These may then be followed by an accessory proteins connecting the inner core with the outer subunits mediating specificity toward the activating agent. These outer subunits are represented by NLR family CARD domain-containing protein 4 (NLRC4), NLR family pyrin domain-containing 3, 6, or 7 (NLRP3/6/7), interferon-inducible protein AIM2 (AIM2), gamma-interferon-inducible protein Ifi-16 (IFI16), or pro-caspases 11, 4, or 5. As a result upon activation, the inner caspases proteolytically activate interleukins 1β and 18 (IL1β/18), mediating inflammation, or gasdermin D, initiating pyroptosis (Zheng et al., 2020). Interestingly, the inflammasome presents an additional step regulated by cholesterol. Efficient activation of the complex machinery by NLRP3 hereby is reliant on cholesterol shuttling to the ER (de La Roche et al., 2018) and enhanced by cholesterol accumulation (Le Bras, 2018). In line with this, cholesterol efflux reduces the activity of the inflammasome (Westerterp et al., 2018). Concordantly, the activation of SREBP-mediated cholesterol synthesis seems to be enhanced by the latter (Im et al., 2011; Li et al., 2013; Guo et al., 2018) and sets LD-mediated, virus-induced inflammatory responses into a fitting context (Negash et al., 2013; Dias et al., 2020).

## Cholesterol in the Endosomal System

**Figure 1:**
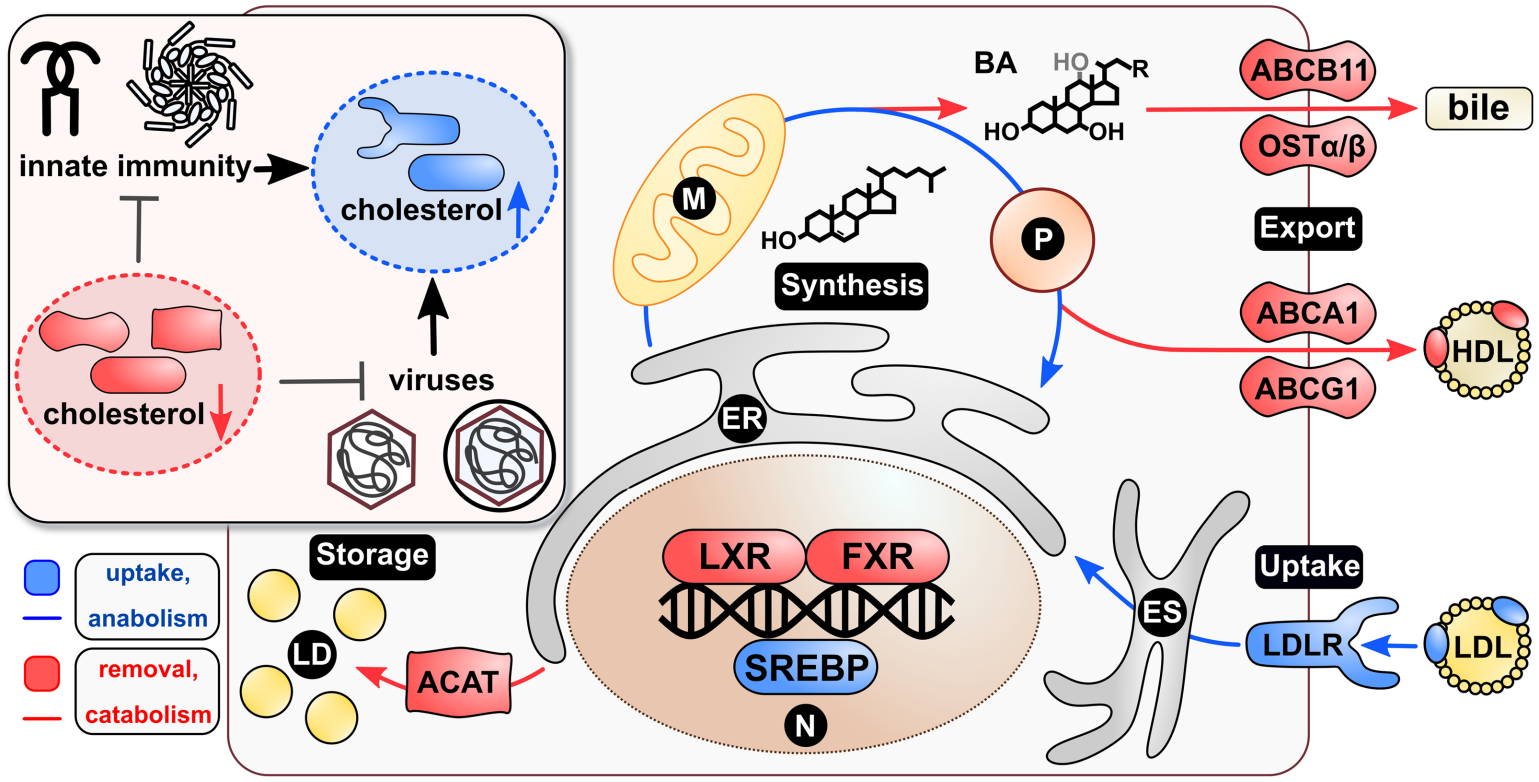
Viruses and innate immunity shift the cholesterol homeostasis to the side of anabolism and uptake. Maintenance of the cellular cholesterol homeostasis is transcriptionally controlled *via* SREBPs and LXR/FXR acting as transcription factors. The first induce uptake and anabolism, whereas the latter induce removal and catabolism of cholesterol. Specifically, SREBPs induce the expression of LDLR leading to cholesterol uptake *via* LDL. This is then shuttled *via* the ES to the ER to be available in the cell. Further, they induce ER-, P-, and M-mediated *de novo* synthesis of the lipid. Once excess cholesterol is present in a cell, LXR induces direct cholesterol exporters, such as ABCA1/G1 leading to HDL synthesis. Secondly, LXR primes P- and M-mediated cholesterol derivatization *via* BA, which are exported by FXR-induced ABCB11 and OSTα/β. A third way of removing cholesterol from a cell is the ACAT-dependent storage of cholesterol esters in LDs. Interestingly, both viruses and innate immunity seem to be inhibited by withdrawal of cellular cholesterol. Similarly, both seem to induce mechanisms resulting in elevated cellular cholesterol, thus leading to a strong intertwinement with respect to the requirement of the lipid. Blue coloring=cholesterol uptake or anabolism; red coloring=cholesterol removal or catabolism. BA, bile acids; ER, endoplasmic reticulum; ES, endosomal system; LD, lipid droplets; M, mitochondrion; P, peroxisome; N, nucleus.

As implied by mechanisms summarized above and in Figure 1, cholesterol affects a variety of steps in the cellular and thereby viral life cycle. Although almost all viruses have an impact on cholesterol metabolism and cholesterol import, there are profound differences with respect to the effect of cholesterol on the viral life cycle. In light of this, it appears rational to affect a central factor involved in cholesterol uptake and intracellular transport as a target for antiviral therapies. One such factor is represented by the endosomal system, which primarily is described as the transporting machinery from components of the extracellular space to intracellular organelles. Primed by endocytosis, early endosomes (EEs) are formed close to the PM. These can either differentiate into recycling endosomes (REs) flowing into the endosomal recycling compartment (ERC), from where cargo is shuttled back to the PM (Xie et al., 2016), or they can mature into late endosomes (LEs; Stoorvogel et al., 1991). On the way from EEs to LEs, the endosomal system can be fed *via* two further routes: (i) vesicles originating from the trans-Golgi network (TGN; Burd and Cullen, 2014; Nagano et al., 2019), aiding in endosomal maturation, and (ii) autophagosomes (Gordon and Seglen, 1988; Ganesan and Cai, 2021), mediating cargo release (Claude-Taupin et al., 2017) or degradation. Further, cargo loading of the endosomal system is mediated by a distinct subset of late endosomes, namely, multivesicular bodies (MVBs). Sorting herein is mediated by the endosomal complexes required for transport (ESCRT) machinery (Frankel and Audhya, 2018), which incorporates molecules into intraluminal vesicles, hence the name MVBs. Cargo, being either protein, nucleic acids or lipids, is then targeted for release *via* exosomes (Hessvik and Llorente, 2018) or degradation *via* fusion with lysosomes (LYs; Mullock et al., 1998).

### Endosomal Flux Is Regulated by Cholesterol Content

Cholesterol fulfils central regulatory roles within the endosomal system. The lipid affects membrane curvature (Chen and Rand, 1997; Lee et al., 2020), thickness (de Meyer and Smit, 2009), fluidity (Cooper, 1978), and lipid microdomains (Sobo et al., 2007). It thereby regulates recruitment of integral and peripheral membrane proteins to lipid rafts and raft-like clusters (Melkonian et al., 1999; Levental et al., 2010; Lorent et al., 2017). This in turn is essential to endosomal flux, as there are continuous vesicular fission and fusion events. The latter are mainly regulated *via* soluble NSF attachment protein receptors (SNAREs). Both clustering and activity hereof is enhanced by cholesterol (Lang et al., 2001; Milovanovic et al., 2015; Stratton et al., 2016), which renders the lipid being intertwined with the fate of vesicular fusion. On the other hand, endosomal cholesterol also regulates and is regulated by dynamin-mediated vesicle fission (Robinet et al., 2006; Anderson et al., 2021) and is required for ESCRT-mediated ILV scission (Kobayashi et al., 2002; Boura et al., 2012; Bissig and Gruenberg, 2013). This strict regulation of membrane fusion and fission by cholesterol is accompanied by directed trafficking of endosomes along microtubules in the process of their maturation. Here, high endosomal cholesterol concentrations leads to recruitment of the dynein/dynactin motor complex and thereby mediates retrograde vesicle transport (Jordens et al., 2001; Thakur et al., 2020). This finally serves the purpose guaranteeing a functional cholesterol trafficking to its destination.

### Cholesterol Distribution Is Mediated *via* the Endosomal System

The endolysosomal systems marks the final destination for endocytosed cholesterol. From here on, the lipid will be re-distributed toward other organelles or toward the place of storage, which requires an intricate combination of shuttling proteins and organelle-organelle adaptor proteins. Once inside LEs or LYs, cholesterol is mobilized with the help of carrier proteins, such as lysosome-associated membrane glycoprotein 2 (LAMP2; Li and Pfeffer, 2016), saposins (Saps; Locatelli-Hoops et al., 2006), lysosomal integral membrane protein-2 (LIMP-2; Heybrock et al., 2019), or the NPC1/2 system (Watari et al., 1999; Infante et al., 2008). Subsequently, cholesterol is distributed to different organelles through organelle-organelle contact sites. Among these are synaptotagmin 7 (Syt7) mediating contacts to peroxisomes (Chu et al., 2015), mitofusin 2 (Mfn2), and vacuolar protein sorting-associated protein 13A (Vps13A) for mitochondria (Daniele et al., 2014; Muñoz-Braceras et al., 2019), NPC1, oxysterol binding protein-like 1A (ORP1L), Rab-interacting lysosomal protein (RILP), StAR-related lipid transfer domain-containing 3 (STARD3) for the endoplasmic reticulum (ER; Rocha et al., 2009; Wilhelm et al., 2017; Höglinger et al., 2019), or STARD4 for the PM (Iaea et al., 2017). This regulation then is essentially guaranteeing delivery of cholesterol to the ER, where it regulates SREBPs and is stored in LDs, to the site of BA synthesis and to ABCA1 exporting cholesterol, as reviewed elsewhere (Luo et al., 2017; Meng et al., 2020).

## Endosomal Cholesterol Trafficking as Shared Feature in Viral Infections

### Viral Attachment and Entry

Primed by the characterization of the envelope of IAV (UHLER and GARD, 1954), it became apparent that cholesterol is a central component in a variety of viral envelopes (Aloia et al., 1993; Funk et al., 2008; Bremer et al., 2009; Merz et al., 2011; Gerl et al., 2012; Carro and Damonte, 2013). On the host side, it aids in endocytosis (Rodal et al., 1999) and therefore is required for viral internalization for a wide range of both enveloped and non-enveloped viruses (Ripa et al., 2021). Apart from endocytosed viruses, the lipid is also required for entry of enveloped viruses directly fusing with the PM, such as HIV (Carter et al., 2009) or Kaposi’s sarcoma-associated herpesvirus (KSHV; Raghu et al., 2007). PM cholesterol therefore serves as first regulator in a viral life cycle, which is either supplied by ER-PM contact sites or ERC-PM contact sites. While various adaptors for ER-PM shuttling have been identified (Naito et al., 2019; Li et al., 2021), ERC-PM contacts present as rather enigmatic although evidently being similarly important (Hölttä-Vuori et al., 2002; Möbius et al., 2003; Garbarino et al., 2012; Iaea et al., 2017). Further elucidation of the latter and how it is modulated by viruses thus presents as important aspect of focus. After cholesterol-dependent steps in endocytosis, shuttling of the lipid within virus-containing endosomes further is important for the uncoating, and therefore for final cellular entry, of enveloped viruses, such as members of the *Filoviridae* family, IAV, HIV, SARS-CoV-2, and Alphaviruses (Schroeder, 2010; Carette et al., 2011; Côté et al., 2011; Garcia-Dorival et al., 2020; Martyna et al., 2020; Sousa et al., 2020). This process is reported to be mediated *via* cholesterol-shuttling proteins, such as NPC1. Besides the latter, also lysosome-associated membrane glycoprotein 1 (LAMP1), which also is capable of binding cholesterol, has been described to facilitate the cellular entry of Lassa virus (LASV; Hulseberg et al., 2018). As these entry factors represent cholesterol-binding proteins and facilitate viral entry, an involvement of the lipid in, for example, facilitating endosomal escape is evident. Advances in how endosomal cholesterol transport machineries affect different viruses therefore also enhances knowledge on possible entry inhibitors.

### Viral Replication

After entry, cholesterol similarly strictly regulates viral replication in manifold cases. For the buildup of the characteristic membranous web around the ER being crucial for HCV replication (Egger et al., 2002; Moradpour et al., 2003), the virus makes use of LE/LYs contact sites and cholesterol trafficking mediated by STARD3, ORP1L, and NPC1 (Stoeck et al., 2018). This recruitment appears to be a similarly important feature for the closely related Flaviviruses (Wang H. et al., 2014; Merino-Ramos et al., 2016; Neufeldt et al., 2018). Although not being known for web formation, the more distantly related CVB3, EMCV and Aichi virus (AiV) seem to share the importance of cholesterol being shuttled to the ER (Albulescu et al., 2015; Ishikawa-Sasaki et al., 2018).

### Viral Morphogenesis and Egress

On the side of viral release, virtually all routes of viral egress, including autophagy-induced cell lysis, strictly depend on membrane fusion or fission events. Efficient cholesterol distribution herein is required for the secretory pathway, the exosomal pathway, or direct budding from the plasma membrane (Wang et al., 2000; Ridsdale et al., 2006; Kobuna et al., 2010; Boura et al., 2012). Here, the main focus of research was set on MVBs and related protein complexes. Blockage of the NPC1/2 system, for example, inhibits MVB-dependent HCV release (Elgner et al., 2016) and the life cycle of both DENV and ZIKV (Poh et al., 2012; Sabino et al., 2019). This similarly could expose weaknesses of other viruses as HBV, hepatitis A virus (HAV), and HEV (Lambert et al., 2007; Hoffmann et al., 2013; Nagashima et al., 2014; González-López et al., 2018) or other enteric viruses (Zhang et al., 2021), as they rely on a comparable release pathway. Apart from MVB-located ESCRT, also the budding process of HIV on PMs needs to be regarded (Sundquist and Kräusslich, 2012). Specifically, the requirement of ESCRT-III renders the need of local cholesterol clustering (Morita et al., 2011; Yandrapalli et al., 2016). Here again, ERC-PM contacts may come into play as discussed above. On the other hand, studies point into the direction that NPC1 and lysosomal recruitment to the cell periphery may be involved in maintaining cholesterol supply to these sites of release (Tang et al., 2009; Cinti et al., 2017). These mechanisms directly link endosomal maturation, which relies on cholesterol, to viral release. Which role lysosomes fulfill in the role of virion production, however, needs to be further studied. Reason for this is growing evidence that some viruses, for example, β-Coronaviruses (Ghosh et al., 2020), make use of these organelles for viral release, therefore bringing cholesterol-regulated endolysosomal maturation into focus.

### Peroxisomes and Mitochondria

Lastly, organelles being majorly involved in the cholesterol metabolism *per se* need to be regarded. Both peroxisomes and mitochondria are essential in anabolism and derivatization of cholesterol (Kovacs et al., 2002). As pointed out earlier, cholesterol has the potential to control innate immunity with respect to IFN responses. Additionally, there is growing awareness that its anabolism and its derivatization into oxysterols or BAs mediate central aspects of inflammation (Spann et al., 2012; Perucha et al., 2019; Willinger, 2019; Cardoso and Perucha, 2021). How and if cholesterol trafficking to these organelles affects these mechanisms remains largely elusive so far. One hint can be found in DENV being reported to cleave Mfns (Yu et al., 2015). This in turn could diminish mitochondrial cholesterol levels and subsequently reduce inflammasome activity (Ichinohe et al., 2013). Elucidation of these processes therefore could present useful with respect to other viruses and their relation to inflammatory responses.

## Conclusion and Outlook

**Figure 2:**
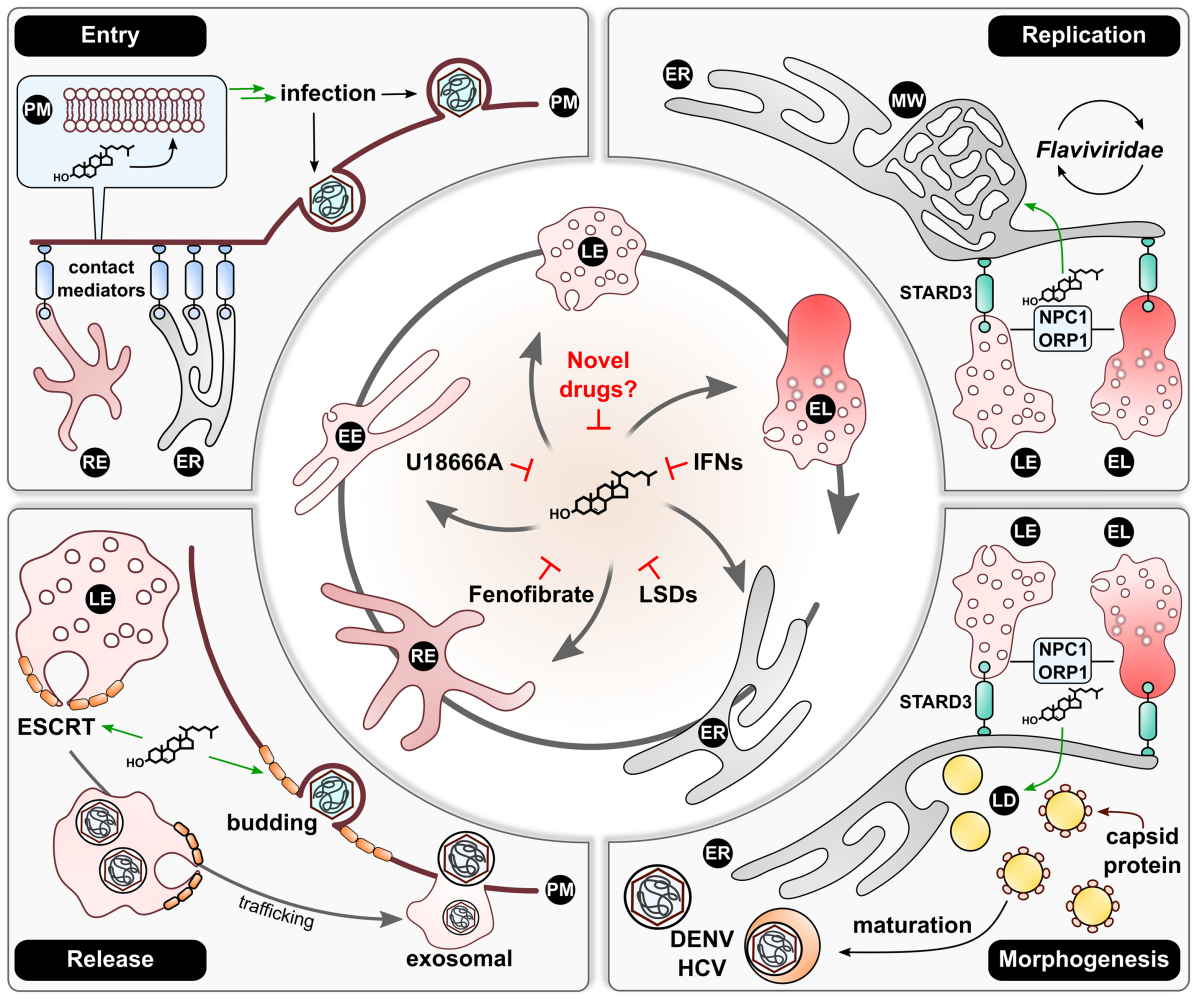
An efficient endosomal re-distribution of cholesterol is required for sustainment of viral life cycles. The endosomal system contains central organelles maintaining cellular cholesterol distribution. Viruses thus make use of these lipid-mobilizing mechanisms to ensure sufficient spatial cholesterol supply during their life cycles. For infection or re-infection, viruses require local cholesterol accumulations at the site of PM fusion or endocytosis. These are fueled by the lipid being transported from the ER or REs, which is dependent on various adaptors establishing respective contact sites to the PM. After infection, especially members of the *Flaviviridae* family were described to induce a cholesterol-enriched MW or replication organelles at the ER. Lipid supply hereby is enhanced by recruiting LEs and ELs to the ER *via* STARD3 with subsequent cholesterol shuttling in an NPC1/ORP1-dependent manner. Similarly, well characterized for this virus family is the dependency on LDs for morphogenesis. Here, DENV and HCV rely on a comparable mechanism involving LEs and ELs for providing ER cholesterol and therefore LD morphogenesis. Lastly, various studies demonstrated that a broad spectrum of viruses is reliant on the ESCRT machinery for viral release either *via* the LE-mediated exosomal route or *via* ESCRT-mediated PM budding. The activity of ESCRT-III stands in close relationship with the local cholesterol concentration, which renders the lipid fulfilling a vital role in the production of viral progeny. Once an efficient cholesterol distribution is perturbed, viral life cycles tend to collapse. This was made use of in the past by inducing cholesterol accumulations by application of U18666A, IFNs, or Fenofibrate. Additionally, the artificial induction of LSDs yielded comparable effects. Thus, this mechanism could present as essential target to novel broad-spectrum antivirals. EE, early endosome; EL, endolysosome; ER, endoplasmic reticulum; IFNs, interferons; LD, lipid droplet; LE, late endosome; LSDs, lysosomal storage disorders; MW, membranous web; RE, recycling endosome; PM, plasma membrane.

As presented above and in Figure 2, viruses indeed seem to rely on a certain level of cholesterol within a cell to ensure an ongoing life cycle and therefore perturb the host metabolome. However, by simply reducing cellular cholesterol in an antiviral approach, one could also potentially dampen the immune system, as evidenced previously (Bahrami et al., 2018; Koike et al., 2021). By having a closer look on cholesterol shuttling within the endosomal system, it becomes apparent that viruses strongly rely on an intact lipid flux. Thus, the key to finding a real pan-antiviral strategy may come down to withdrawing cholesterol from where it is needed for viral maintenance rather than reducing it globally. This very mechanism could be achieved by inducing a deliberate accumulation of cholesterol within the endosomal system. Here, recent studies identified this as restrictive action against IAV in the context of an IFN response (Kühnl et al., 2018) and against HEV as a consequence of heavy cholesterol uptake and Fenofibrate application (Glitscher et al., 2021). Similarly, this could explain the broad antiviral effect of lysosomotropic drugs, such as U18666A (Kuzu et al., 2017). While the tools at hand already present promising treatment options against viral infections, more research is required in assessing adverse effects such as lysosomal storage diseases. Thus, advances in clarifying molecular mechanisms and regulators in endosomal cholesterol shuttling may finally lead to the discovery of a tolerable, pan-antiviral therapy.

## Author Contributions

EH and MG: conceptualization. MG: writing – original draft preparation and visualization. EH: writing – review and editing, supervision, project administration, and funding acquisition. All authors contributed to the article and approved the submitted version.

## Funding

This work was supported with a grant by the LOEWE Center DRUID (Novel Drug Targets against Poverty-Related and Neglected Tropical Infectious Diseases; project D2).

## Conflict of Interest

The authors declare that the research was conducted in the absence of any commercial or financial relationships that could be construed as a potential conflict of interest.

## Publisher’s Note

All claims expressed in this article are solely those of the authors and do not necessarily represent those of their affiliated organizations, or those of the publisher, the editors and the reviewers. Any product that may be evaluated in this article, or claim that may be made by its manufacturer, is not guaranteed or endorsed by the publisher.
